# Phenotypic and Target-Directed Screening Yields New Acaricidal Alternatives for the Control of Ticks

**DOI:** 10.3390/molecules27248863

**Published:** 2022-12-13

**Authors:** Tatiana Saporiti, Mauricio Cabrera, Josefina Bentancur, María Elisa Ferrari, Nallely Cabrera, Ruy Pérez-Montfort, Francisco J. Aguirre-Crespo, Jorge Gil, Ulises Cuore, Dimitris Matiadis, Marina Sagnou, Guzmán Alvarez

**Affiliations:** 1 Laboratorio de Moléculas Bioactivas, Departamento de Ciencias Biológicas, CENUR Litoral Norte, Universidad de la República, Paysandú 60000, Uruguay; 2 Departamento de Bioquímica y Biología Estructural, Instituto de Fisiología Celular, Universidad Nacional Autónoma de México, Ciudad de Mexico 04510, Mexico; 3 Facultad de Ciencias Químico Biológicas, Universidad Autónoma de Campeche, Campeche 24039, Mexico; 4 Laboratorio de Reproducción Animal, Producción y Reproducción de Rumiantes, Departamento de Ciencias Biológicas, CENUR Litoral Norte, Universidad de la República, Paysandú 60000, Uruguay; 5 División de Laboratorios Veterinarios “Miguel C. Rubino”, Ministerio de Ganadería, Agricultura y Pesca, Montevideo 91600, Uruguay; 6 National Center for Scientific Research ‘Demokritos’, Institute of Biosciences & Applications, 15310 Athens, Greece

**Keywords:** acaricide development, cattle tick, drug discovery, triosephosphate isomerase

## Abstract

*Rhipicephalus microplus*, the “common cattle tick”, is the most important ectoparasite in livestock worldwide due to the economic and health losses it produces. This tick is a vector for pathogens of several tick-borne diseases. In Latin American countries, damages reach approximately USD 500 million annually due to tick infections, as well as tick-borne diseases. Currently, resistant populations for every chemical group of acaricides have been reported, posing a serious problem for tick control. This study aims to find new alternatives for controlling resistant ticks with compounds derived from small synthetic organic molecules and natural origins. Using BME26 embryonic cells, we performed phenotypic screening of 44 natural extracts from 10 Mexican plants used in traditional medicine, and 33 compounds selected from our chemical collection. We found 10 extracts and 13 compounds that inhibited cell growth by 50% at 50 µg/mL and 100 µM, respectively; the dose-response profile of two of them was characterized, and these compounds were assayed in vitro against different life stages of *Rhipicephalus microplus*. We also performed a target-directed screening of the activity of triosephosphate isomerase, using 86 compounds selected from our chemical collection. In this collection, we found the most potent and selective inhibitor of tick triosephosphate isomerase reported until now. Two other compounds had a potent acaricidal effect in vitro using adults and larvae when compared with other acaricides such as ivermectin and Amitraz. Those compounds were also selective to the ticks compared with the cytotoxicity in mammalian cells like macrophages or bovine spermatozoids. They also had a good toxicological profile, resulting in promising acaricidal compounds for tick control in cattle raising.

## 1. Introduction

South American countries are the main producers and suppliers of beef internationally [1]; Brazil is the world’s leading exporter of beef, with 200 million heads, and breeding which has increased approximately 30% in the last decade [2]; therefore, any event that damages livestock production in these countries is of high importance and has an economic impact [3,4]. The tick *Rhipicephalus microplus* (*R. microplus*) is one of the main ectoparasites of cattle, generating economic losses in Uruguay of USD 46 million annually, due to production losses, treatments costs, and deaths due to tick-borne diseases [2]. *R. microplus* is a vector of multiple hemoparasites such as *Babesia bovis*, *B. bigemina,* and *Anaplasma marginale,* causative agents of tick-borne diseases. This infection has a fatality rate of approximately 10% [5,6].

Resistance has become more difficult since the emergence of multi-resistant populations in 2009 [7]. It has been observed over time that, as resistance to a certain drug group develops, the drug group is substituted by another compound, to achieve greater efficacy in treatments [8,9]. This not only leads to resistance problems but also to contamination of the environment, risk of direct contamination of operators, and the risk of residues in food of livestock origin [10]. Furthermore, some other chemical groups are used, not only for controlling ticks but also for other parasites [11,12,13,14]. This leads to exposing ticks to subtoxic doses of these drugs, increasing the risk of generating cross-resistance between active principles of the same chemical group [15,16]. In addition, the low molecular diversity of commercial acaricides (only five chemical groups) increases the probability of cross-resistance [17]. We now find resistance in all of the world’s leading exporters of beef.

The research and development (R&D) of new acaricides is of interest to the pharmaceutical industry [17], which has been focusing on the development of different combinations of existing active drugs. Currently, one obstacle is the determination of the maximum limits allowed in foods, and their acceptance by international organizations. It is estimated that in the immediate and medium-term future there will be no new molecules from the pharmaceutical industry available for tick control [17]; thus, R&D for new acaricidal molecules against *R. microplus* is vital.

To discover new acaricidal molecules, in this work we applied two strategies widely used in drug development: phenotypic screening and targeted molecular screening. For the targeted molecular screening, we used triosephosphate isomerase (TIM), which is a glycolytic and gluconeogenic enzyme that catalyzes glyceraldehyde 3-phosphate and dihydroxyacetone phosphate interconversion. Several publications have reported the potential of TIM as a suitable target for drug development against various parasites that cause human diseases, such as *Plasmodium falciparum*, *Trypanosoma cruzi*, *Trypanosoma brucei*, *Fasciola hepatica*, and *Giardia lamblia* [18,19,20,21]. Although the structural similarity of this enzyme is highly conserved between species, it is possible to obtain selective inhibitors as long as they act at the interface of the enzyme, as this is a part of the enzyme which is poorly conserved between species [22]. For the phenotypic screening, we used the only insolated cells from susceptible *R. microplus* available in culture, derived from tick embryos. In this work, we started the process of discovering new acaricides using phenotypic and target-directed screening of molecules from synthetic and natural origins. We explored acaricidal activities and performed some preclinical studies with some of these molecules.

## 2. Materials and Methods

### 2.1. Chemical Collection and Natural Extracts

A natural product collection was generated based on human traditional medicinal use. We selected 10 plants from southeastern Mexico (Caribbean region) from more than 300 species used in traditional Mayan medical practice (Table S1 in the Supplementary Materials) because of the availability and abundance at the time of collection. These plants were chosen following the indications of the “chamanes” (the traditional Mayan physicians), and were then classified taxonomically by botanists. A robust system was used to maximize the extraction of stable molecules. Methanol was used to extract the most hydrosoluble compounds, and dichloromethane was used to extract the most lipophilic components. High temperature was used in some steps to enhance solubilization [23], as shown in Table S1 in the Supplementary Materials. We used 44 extracts from 10 species in the phenotypic screening.

The synthetic compounds were selected from an in-house library belonging to the Universidad de la República, Uruguay. Compounds **Mar105**, **Mar 106**, **DM97**, and **DM83** were kindly provided by the NCSR “Demokritos” group. Compounds **Mar105**, **DM97,** and **DM83** were prepared according to previously published procedures [24,25]. The synthesis of **Mar106** is described in the Supplementary Materials. These molecules belong to a chemical collection with a diversity of structures, classified into different families including benzofuroxanes, chalcones, diarylideneketones, flavones, thiazoles, thioureas, steroids, thiadiazines, indazoles, and hydrazines (Supplementary Table S2) [26,27,28,29]. We selected 33 compounds from our collection for the phenotypic screening because of their antiparasitic activity in other parasites, and 86 compounds were selected for the target-directed screening using TIM, on the basis of their structural relationship with the reported TIM inhibitors.

### 2.2. Phenotypic Screening

#### 2.2.1. Effect of Compounds on BME26 Cell Cultures

The embryonic cell line BME26 from *R. microplus* (Centro de Biotecnologia, Universidade Federal do Rio Grande do Sul, Porto Alegre, RS, Brazil) was grown as adherent monolayers in a complete medium, as described previously [30], which consisted of L-15B300 medium supplemented with 5% heat-inactivated Fetal Bovine Serum (FBS) (Gibco Co., Grand Island, NY), 10% Tryptose Phosphate Broth (TPB) (BD), penicillin (100 U/mL), streptomycin (100 mg/mL, Gibco Co., Grand Island, NY, USA), and 0.1% bovine lipoprotein concentrate (ICN), (pH 7.2). The suspension of embryonic BME26 cells from *R. microplus* was seeded into 24-well plates (5 × 10^5^ cells/well) to a final volume of 500 μL in a complete medium, and allowed to attach. After 24 h incubation at 34 °C, the extracts (100 µg/mL final concentration) and the compounds were added to the final concentrations indicated (1–100 µM), and 0.05% dimethylsulfoxide (DMSO) was used in the negative control wells. After 24 h of treatment, 50 μL of 3-(4,5-dimethylthiazol-2-yl)-2,5-diphenyltetrazolium bromide (MTT) prepared in serum-free medium (5 mg/mL) was added to each well. The media were discarded after 2 additional hours of incubation, and 1 mL of acid-isopropyl alcohol (0.15% HCl in isopropyl alcohol) was added to dissolve the formazan crystals. The mixture was transferred to 1.5 mL tubes, centrifuged at 6000× *g* for 15 min, and the clear supernatant was collected in new tubes for absorbance measurement at 570 nm using quartz cuvettes in a UVmini-1240 UV–vis spectrophotometer (Shimadzu, Japan). Absorbance values of the control treatment were used for normalization (100% viability). The activity was determined by analysis using OriginLab8.5^®^ sigmoidal regression (% of viable cells compared to the logarithm of the compound concentration). The half-maximal inhibitory concentration (IC_50_), defined as the drug concentration at which 50% of the cells were viable relative to the control (no drug added), was used as a measurement of activity. These experiments were performed in triplicate for each compound.

#### 2.2.2. Effect of Compounds on BME26 Cell Morphology [31]

The BME26 cells (5 × 10^5^ cells/well) were plated on glass coverslips (Corning^®^ Costar^®^, Cambridge, MA, USA), then introduced into 24 well plates and incubated in a complete medium, to attach at 34 °C for 24 h. Chemical inhibitors were added at the final concentrations indicated in the cell culture experiments, and 0.05% DMSO was used in the negative control wells. After 24 h of treatment, the cells were washed with 0.15 M NaCl, 10 mM sodium phosphate, and pH 7.2 (PBS), and immediately fixed in a buffered 4% formaldehyde solution (PBS) for 15 min at room temperature (RT). The cells were then incubated for 20 min (RT) in 200 μL of a solution containing the nuclear marker DAPI (4,6-diamidino-2-phenylindole, dihydrochloride, Molecular Probes, D1306-1 μg/mL) and 1 μL of the F-actin probe phalloidin (Alexa Fluor^®^ 555, Molecular Probes, A34055-300 units). The cells were visualized using a Leica DMI4000 inverted fluorescent microscope equipped with two A4 (DAPI) filter cubes and a N2.1 (Phalloidin) filter.

### 2.3. Effect of Compounds on the Enzymatic Activity of TIM

*R. microplus* triosephosphate isomerase (RmTIM) and *Homo sapiens* triosephosphate isomerase (HsTIM) were expressed in *Escherichia coli*, and purified as previously described in the literature [31]. Protein concentration was determined by absorbance measurements at 280 nm for RmTIM (ε = 33440 M^−1^cm^−1^) and HsTIM (ε = 33460 M^−1^cm^−1^). Enzymatic activity was determined following the reaction of the conversion of glyceraldehyde-3-phosphate to dihydroxyacetone phosphate in a coupled enzyme assay using α-glycerol-phosphate dehydrogenase. The decrease in absorbance at 340 nm was recorded in a multiplate reader Varioskan™ Flash Multimode Reader (Thermo Scientific^TM^, Waltham, MA, USA) at 25 °C. The reaction mixture (1 mL, pH 7.4) contained 100 mM triethanolamine, 10 mM EDTA, 0.2 mM NADH, 1 mM glyceraldehyde-3-phosphate, and 0.9 units of α-glycerol-phosphate dehydrogenase. The reaction was initiated by the addition of 5 ng/mL of the TIM of interest. For the inhibition studies (incubation mixture), TIM was incubated at a concentration of 5 mg/mL in a buffer of pH 7.4 containing 100 mM triethanolamine, 10 mM EDTA, and 10% DMSO at 25 °C for 1 h. The mixture also contained the compounds, dissolved in DMSO, at final concentrations of 10 and 100 μM. After 1 h, 10 μL of the incubation mixture was added to the reaction mixture, to a final volume of 100 μL in a 96-well plate. None of the molecules tested here affected the activity of α-glycerol-phosphate dehydrogenase (checked in situ on the reaction mixture inhibited, with the addition of fresh α-glycerol-phosphate dehydrogenase and running the measurement again). The IC_50_ value was taken as the concentration of drug needed to reduce the enzymatic activity by 50%, and was analyzed using OriginLab 8.5 Corporation, Northampton, MA, USA, sigmoidal regression (% of enzymatic activity vs. the logarithm of the compound concentration). The tests were performed in triplicate in two independent experiments.

### 2.4. Nonspecific Cytotoxicity

#### 2.4.1. Cytotoxicity Assay on Murine Macrophages

J774.1 murine macrophages (ATCC, Rockville, MD, USA) were grown in a DMEM culture medium containing 4 mM L-glutamine, and supplemented with 10% FCS. The cells were seeded into a 96-well plate (5 × 10^4^ cells in 200 µL culture medium) and incubated at 37 °C in a 5% CO_2_ atmosphere for 48 h, to allow cell adhesion before drug testing. Afterward, the cells were exposed for 48 h to the compounds (25–400 μM) or the vehicle (0.4% DMSO), and additional controls (cells in medium) were used in each test. Cell viability was then assessed by measuring the mitochondria-dependent reduction of MTT to formazan: for this, MTT in sterile PBS (0.2% glucose), pH 7.4, was added to the macrophages, to a final concentration of 0.1 mg/mL, and the cells were then incubated at 37 °C for 3 h. After removing the medium, the formazan crystals were dissolved in 180 μL of DMSO and 20 μL of MTT buffer (0.1 M glycine, 0.1 M NaCl, 0.5 mM EDTA, pH 10.5), and the absorbance was measured at 560 nm. The IC_50_ was defined as the drug concentration at which 50% of the cells were viable, relative to the control (no drug added), and was determined by analysis using OriginLab 8.5 Corporation, Northampton, MA, USA, sigmoidal regression (% of viable cells compared to the logarithm of the compound concentration). The tests were performed in triplicate in two independent experiments [32].

#### 2.4.2. Cytotoxicity Assay on Bovine Spermatozoa

Semen samples were obtained from a sperm bank (Gensur Ltda. Montevideo Uruguay), and were kept under liquid nitrogen until use. The semen used belonged to a single freezing batch that was obtained during a regular collection schedule with an artificial vagina. Samples from three straws were thawed, and a sperm pool was prepared in PBS at a concentration of 40 million spermatozoa per mL; then, 50 μL of this sperm suspension was carefully mixed with 50 μL of compounds diluted to 100 and 50 μM or with 1% *v*/*v* DMSO in control experiments. Each condition was assayed in 96-well plates in duplicate, and controls were assayed in triplicate. Plates were incubated at 37 °C for 1 h with moderate shaking. The motility analysis was carried out using a CASA (Computer-Assisted Semen Analyzer) system Androvision (Minitube, Tiefenbach, Germany) with an OlympusBX 41 microscope (Olympus, Japan) equipped with a warm stage at 37 °C. Each sample (10 μL) was placed onto a Makler Counting Chamber (depth 10 μm, Sefi-Medical Instruments, Haifa, Israel), and the following parameters were evaluated: percentage of total motile spermatozoa (motility > 5 μm/s) and curve line speed (CLS > 24 μm/s). At least 400 spermatozoa were analyzed from each sample, from at least four microscopic fields. For each dose of the compound, the toxic effect was compared to the control (1% DMSO).

### 2.5. Adult Immersion Test (AIT)

This essay was performed based on Drummond et al., 1973, as each group of 5 fully engorged females of the Mozo susceptible strain of *R. microplus* was immersed in 50 mL tubes containing 10 mL of compounds at different concentrations (0.5, 1, 1.5, 2, 3.5 mM) for 15 min in four replicates. Four negative control groups were used (DMSO 10%). After immersion, the fully engorged females were lightly dried with absorbent paper. They were then placed in 100 mm disposable Petri dishes with their respective identification, and immediately incubated at 27 °C in an atmosphere with relative humidity (RH) of 85-90% for 14 days. After this period, the females that did not lay eggs were considered dead. The females that did lay eggs were discarded, while their eggs were weighed and transferred to glass tubes which had a manually compressed cotton plug humidified with 2 mL of distilled water at their bottom. The tubes carrying the eggs were closed with hydrophilic cotton, and were returned to the incubator chamber under the same conditions until the eggs hatched. The reproductive efficacy, as well as % control of each compound on *R. microplus*, was calculated according to published reports [33]. The results were verified in two independent experiments.

### 2.6. Larval Immersion Test (LIT)

This assay was performed based on the method of Castro-Janer et al., 2011. Fully engorged females of *R. microplus* (Mozo strain) were incubated in Petri dishes at 27 °C and 85–90% relative humidity for 14 days. After this period, the hatched eggs were collected and placed in glass tubes that had a manually compressed cotton plug humidified with 2 mL of distilled water at their bottom, and were later closed with a hydrophilic cotton plug. The tubes were incubated in the same conditions as described above. The larvae were ready for testing after 14 to 21 days from egg-hatching. For the test, the compounds were diluted in DMSO, and were later resuspended in distilled water, always using 15% DMSO of the final solution volume. The concentrations for the different compounds for the final immersion solution are 1 mg/mL for the extracts, and 1 mM for the compounds. A solution of 15% of DMSO in distilled water was used as control, and another solution of commercial ivermectin (Sanimax, Laboratorio Adler, Montevideo, Uruguay) was diluted in distilled water to a concentration of 0.082 mM (described as the CL 99% for Mozo [34]). For the immersion procedure, tubes containing the larvae were placed vertically, without the cotton plug, and the larvae were taken with a brush from the top of the tube, ensuring they were viable. A cluster with a diameter of 2 mm of larvae was collected with a brush, and was later immersed in each solution, as well as in both control solutions, for 10 min. After this time, the solutions were drained, and the larvae were placed in 8.5 cm × 7.5 cm Whatman No. 1 filter papers. The filters were folded in the middle of the 8.5 cm side, and closed with 3 bulldog clips, forming packets. These packets were incubated for 24 h, under the same conditions as described previously, and then the percent of mortality was determined, considering it as the % of the control.

### 2.7. Toxicology and Pharmacokinetic Profiles

The predictions were made with open-access SwissADME software (http://www.swissadme.ch accessed on 1 September 2021), a tool that allows the prediction of different pharmacokinetic parameters such as water solubility, gastrointestinal absorption, skin penetrability, lipophilicity, bioavailability, etc., (details in the Supplementary Materials) and T.E.S.T (Toxicity Estimation Software Tool). The software input used the SMILES codes of the molecules, which were generated with ChemBioOffice 2010 software. We also made an exhaustive revision of the relevant chemical information of the compounds in the PubChem database (until September 2021).

## 3. Results

**Figure 1:**
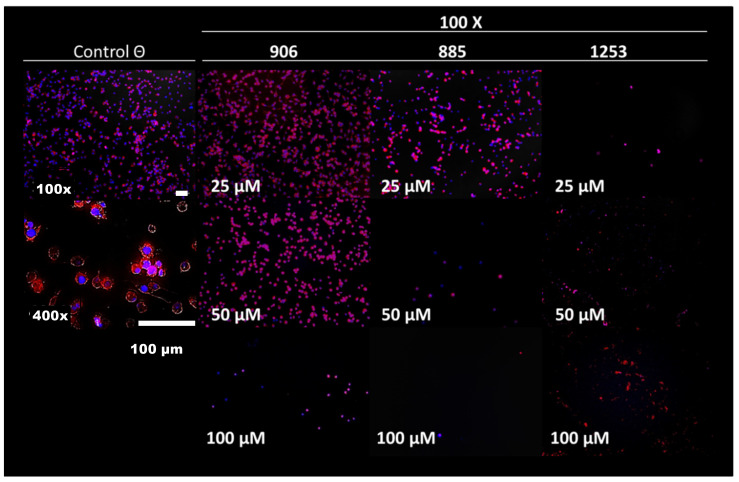
Dose–response profile of compounds **906, 885,** and **1253**. Confocal fluorescent microscopy of BME26 cells incubated for 24 h with different concentrations of the compounds (25, 50, and 100 μM, at 100× magnifications). Following incubation, cells were stained with DAPI and phalloidin to observe the cell architecture (at 100× and 400× magnifications, the white bars on the bottom, indicates the 100 µm scale size).

Forty-four extracts from 10 different species of plants from the southeastern area of Mexico were assayed on embryonic cells (line BME26) from a susceptible *R. microplus*, at a concentration of 100 μg/mL. Eleven of the extracts inhibited more than 50% of cell growth (Table 1). Thirty-four compounds from our chemical collection were also assayed with the same cells, at a concentration of 100 μM (Table S2 in the Supplementary Materials). Thirteen of the compounds inhibited more than 50% of cell growth under these conditions (Table 2). The IC_50_ values were: 24 ± 3 µM for compound **906**; 17 ± 4 µM for compound **885**; 12 ± 1 µM for compound **1253;** 20 ± 3 for compound **795**; and 15 ± 3 µM for compound **796**. A dose–response profile using microscopy for three of these compounds is shown in Figure 1: in this figure, there are no cells at 25 µM consistent with those observed by the MTT assay. The viability of the cells is shown at 400x with a coloration of the cytosol and the nucleus.

Table 3 summarizes the results of the target-directed screening at 10 μM or 100 μM concentration of synthetic compounds against RmTIM and HsTIM. Compounds **910**, **1367, 1404, 799, 1387, 1385**, **1386**, **879**, **Mar105**, **Mar106**, and **DM83** exhibited considerable inhibitory activity, with IC_50_ values between 0.3 and 25 µM. It is also important to note that HsTIM was not inhibited by these compounds at 10 μM, suggesting species-specific enzyme inhibition.

The acaricidal activity on *R. microplus* was measured using the AIT at 500 µg/mL for the plant extracts (Table 4). The synthetic compounds were used for the LIT and the AIT, at 1 mM and 1.5 mM, respectively (Table 4). Of the 11 extracts assayed, 4 were active, showing > 50% of tick mortality: these were the methanolic and the dichloromethanolic extract from *A. hispida* (**T15** and **T26**, respectively)*,* the dichloromethanolic extract from *B. crassifolia* (**T28**), and the methanolic extract from *R. nudiflora* (**T44**) in AIT. The synthetic chemical collection had five compounds with acaricidal potential: **906**, **795, 1253, DM83,** and **885**. Compounds **910**, **1367, 1404, 799, 1387, 1385**, **1386**, **879**, **Mar105**, and **Mar106** did not show reproducible acaricidal activity.

We also reviewed and performed theoretical calculations on the synthetic compounds which had shown the best results in our experiments, to predict some of their toxicological and pharmacokinetic parameters (Table 5). Mutagenicity and oral acute toxicity are parameters recommended by the FDA to predict in vivo toxicity problems. Solubility, lipophilicity, metabolic stability, and skin permeability characterize the pharmacokinetic behavior of a substance in vivo. The in vitro selectivity of compounds **885** and **DM83** at 50 μM was compared using murine macrophages (Table 2) and bovine sperm. None of those compounds revealed cytotoxicity at this concentration (data not shown).

## 4. Discussion

Of the 10 different medicinal plant species used in this work, 3 of them demonstrated acaricidal potential (*A. hispida* (**T15** and **T26**), *B. crassifolia* (**T28**), and *R. nudiflora* (**T44**)). Some of the major compounds that have been isolated from *A. hispida* are [35]: sesquiterpene lactones ambrosin, damsin, flavone hispidulin, and from *B. crassifolia* [36]: 5-O-galloylquinic acid, 3-O-galloylquinic acid, 3,4-di-O-galloylquinic acid, 3,5-di-O-galloylquinic acid, 3,4,5-tri-O-galloylquinic acid, and (+)-epicatechin-3-gallate. None of those were reported as acaricidal compounds, but some of them are structurally similar to **885**, a chalcone derivate like 3-O-caffeoylquinic acid. *R. nudiflora* has not been studied further, so no major components have been identified. The use of natural extract directly in the field has limitations regarding the stability and concentration of the components. These extracts will be used in a future bio-guided fractionation, to identify the specific acaricidal components and subsequently, using rational design, modify those molecules to get better acaricidal compounds.

The phenotypic screening yielded 15% of hits, i.e., five cytotoxic compounds for tick cells. The target-directed screening yielded 13% of HITs, i.e., 11 compounds which inhibited RmTIM activity. Those inhibitors were also species-selective, and one was more potent than the others previously reported [31]. **DM83** was the most effective and, interestingly, the organic ligand was inactive against RmTIM. This difference indicates a high selectivity and specificity potential for this compound.

**Figure 2:**
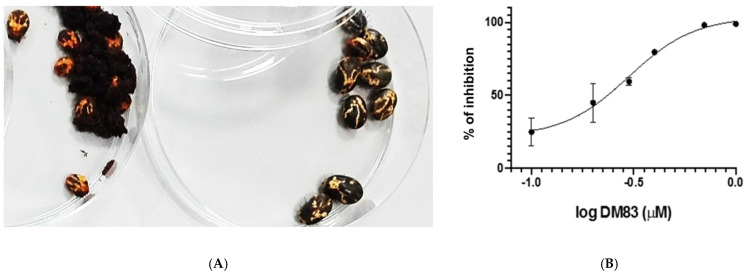
(**A**) Picture showing day 14 of the AIT. To the left, the non-treated ticks and, on the right plaque, ticks treated with 1.5 mM **885**; in this case, 100% of the ticks were dead. (**B**) Dose–response curve of compound **DM83** on the activity of RmTIM. Error bars represent standard deviation and n = 3 for each concentration.

From hundreds of synthetic compounds in our chemical collection, we found five promising molecules. Compound **1253** caused an effect only at the larval stage, with 50% mortality whereas compound **795** exhibited a significant acaricidal effect, causing 70% mortality in the AIT. Compound **796,** on the other hand, exhibited weak activity in both the LIT and AIT, causing 36 and 20% mortality, respectively. Similarly, compounds **DM83** and **885** had considerable effect in both assays. More specifically, for **DM83** the % mortality measured for the LIT assay was 69% and, for the AIT, 50%. Compound **885** was the most potent, with an activity of 100% in the LIT test and an LC_50_ of 1 mM in the AIT assay (Table 4 and Figure 2). In addition, this compound, in a preliminary study with a multiresistant strain, had acaricidal activity (data in supporting material). Compounds **906**, **796**, **266**, **DM83,** and **885** all had acaricidal potential against *R. microplus* similar to Amitraz (which is widely used for tick control), and with quite a safe toxicology profile. Compound **906**, known as curcumin, is commonly used in human food preparation, and has a long history as a potential treatment for a variety of human diseases; the use of this compound for tick control would be very safe and environmentally friendly; it could be used directly in a clinical trial with cattle, after the formulation, application, and dosage had been explored. On the other hand, compounds **796**, **795,** and **885** are in the early stages of the drug development process. These compounds need to be studied more extensively, but their toxicology profile seems to be safe. Compounds **796** and **795** are molecules with simple and low-cost production. Compound **DM83** has been the most potent and selective RmTIM inhibitor reported until now (Figure 2), and also had acaricidal activity without toxic effect for bovine sperm and murine macrophages at 50 µM. The rest of the RmTIM inhibitors did not demonstrate acaricidal activity because of their solubility in the acaricidal assay, indicating that a formulation based on lipidic components could be better than DMSO. All the studied compounds need to be extensively further studied in different formulations suitable to be used in cattle, and their acaricidal potency must be evaluated in field studies. In addition, all of these compounds are biodegradable, minimizing residue problems in food derivates, and are also environmentally friendly [37].

This study was situated in the early stage of drug development; more toxicology studies and pharmacokinetic studies on the mechanism of action of some of the drugs are necessary. Before solving the efficacy problem, we need to change some strategies at the discovery stage. The methods used to observe acaricidal activity were not the best, and there were significant problems related to solubility and penetration of the compounds into the tick. The ticks were more than 90% hermetic—those did not permit penetration of the liquids. Ensuring that the drug penetrated the tick presented a major challenge. In addition, we were working in vehicles of these compounds; we know that commercial compounds like amitraz are prepared in a special oil emulsion; when the tick comes in contact with this solution, it stays in them for a long time. There are other methods, such as artificial feeding of the tick, using blood, but while this method could be used to check the efficacy, it is time- and cost-consuming. There are also significant problems with DMSO; however, we will perform these studies in future.

## 5. Conclusions

We identified five synthetic compounds that could be developed as acaricidal compounds for the control of *R. microplus*, using two different strategies: phenotypic and target-directed screening. The target-directed screening yielded more hits than the phenotypic screening. **DM83** is the most potent and selective inhibitor of tick triosephosphate isomerase described until now. All the compounds are safe, biodegradable, and low-cost: these are important characteristics in veterinary drugs. We also identified three different species of plants with acaricidal potential.

## Figures and Tables

**Table 1:** Phenotypic screening at 100 μg/mL of Mayan medicinal plants on embryonic cell line BME26 from *R. microplus.*

Collection Code	Scientific Name/(Spanish or Mayan Name)/Tissues Employed/Solvent	% of Growth Inhibition	% of Growth Inhibition for Mammalian Cells [23]	Plant Visualization
T2	*Leucaena leucocephala* (Huachi in Mayan) leaves and branches by MeOH	70 ± 7	80	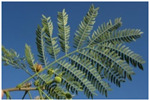 1
T8	*Leucaena leucocephala* (Huachi in Mayan) leaves and branches by CH_2_Cl_2_	72 ± 8	80	
T3	*Cnidoscolus chayamansa* (Chaya in Mayan) leaves by MeOH	63 ± 5	25	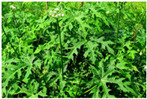
T4	*Cnidoscolus chayamansa* (Chaya in Mayan) leaves by CH_2_Cl_2_	100 ± 9	75	
T25	*Ipomoea pes-caprae* (Riñonera in Spanish) leaves and branches by MeOH	70 ± 7	0	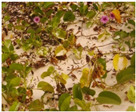
T15	*Ambrosia hispida* (K’an lool xiiw in Mayan) leaves and branches by MeOH	93 ± 5	ND	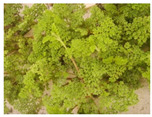
T26	*Ambrosia hispida* (K’an lool xiiw in Mayan) leaves and branches by CH_2_Cl_2_	47 ± 4	ND	
T19	*Malmea depressa* (Elemuy in Mayan) leaves and branches by MeOH	63 ± 7	75	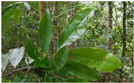 2
T22	*Cecropia obtusifolia* (Guarumbo in Mayan) leaves by CH_2_Cl_2_	100 ± 9	0	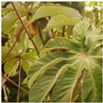
T28	*Byrsonima crassifolia* (Nance in Mayan) tree bark by CH_2_Cl_2_	48 ± 7	50	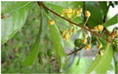 3
T44	*Ruellia nudiflora (Engelm. & A. Gray) Urb.* (Xana mukuy in Mayan) leaves and branches by MeOH	100 ± 8	ND	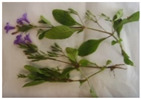

Picture from: 1-Comisión Nacional para el Conocimiento y Uso de la Biodiversidad (CONABIO) 2-IREKANI, Instituto de Biología—UNAM 3-Flora de la Península de Yucatán Herbario CICY, Unidad de Recursos Naturales. ND: not determined.

**Table 2:** Phenotypic screening of synthetic compounds at a concentration of 100 μM using the embryonic cell line BME26 from *R. microplus.*

Chemical Collection Code	Structure	% of Growth Inhibition	IC_50_ (µM)	IC_50_ (µM) Mammalian Cells
**906**	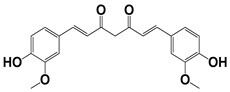	95	24 ± 3	<25
**795**	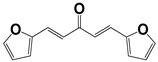	80	20 ± 3	>50
**796**	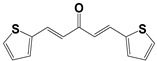	93	15 ± 3	>50
**809**	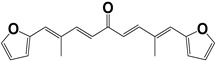	50	>100	>50
**133**	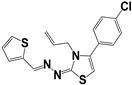	71	>50	>50
**266**	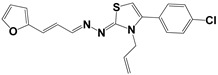	77	>100	>50
**903**	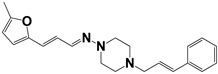	75	>50	>50
**912**	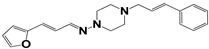	52	>50	>50
**715**	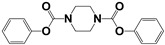	64	>50	ND
**183**	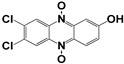	62	ND	<25
**181**	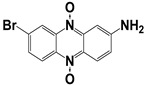	90	ND	<25
**885**	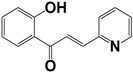	91	17 ± 4	>50
**1253**	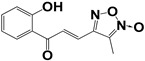	95	12 ± 1	>50

ND: not determined.

**Table 3:** Target-directed screening of synthetic compounds at 10 μM (*) or 100 μM (**) against RmTIM and HsTIM. EC_50_ (µM) cytotoxicity for mammalian cells (murine macrophages). ND: not determined.

Chemical Collection Code	Structure	% Inhibition of Enzymatic Activity of RmTIM at 10 µM * or 100 µM **	IC_50_ (µM)	% Inhibition of Enzymatic Activity of HsTIM at 10 µM	EC_50_ (µM) for Mammalian Cells
**910**	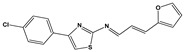	60 **	23 ± 2	0	>50
**1367**	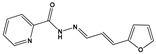	82 *	<10	0	>50
**1366**	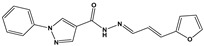	38 **	ND	ND	ND
**1378**	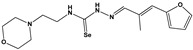	77 **	30	0	ND
**1404**	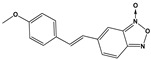	100 *	<10	ND	>50
**799**	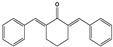	88 *	<10	0	>50
**1387**	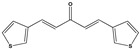	100 *	<10	0	>50
**1088**	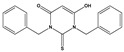	50	>50	ND	ND
**1408**	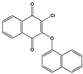	72 **	>50	ND	ND
**1385**	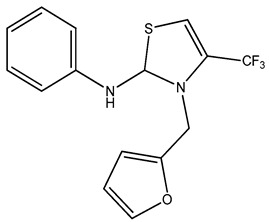	89 *	<10	ND	>50
**1386**	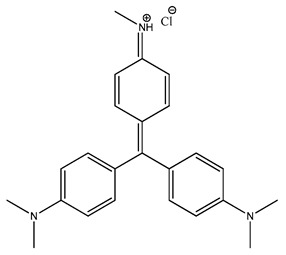	100 *	<10	ND	ND
**879**	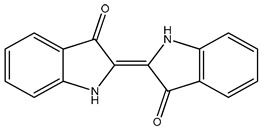	95 *	<10	0	>50
**Mar106**	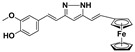	69 **	25 ± 4	0	>25
**Mar105**	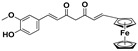	65	10 ± 1	0	>25
**Dm97**	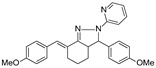	0 **	ND	ND	ND
**Dm83**	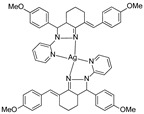	99 *	0.30 ± 0.05	0	>50

**Table 4:** Toxicity effect of extracts (1 mg/mL) and compounds in the LIT (1.0 mM) and the AIT (1.5 mM).

Collection Code	% of Mortality LIT	% of Mortality AIT
**T2**	ND	30 ± 8
**T8**	ND	42 ± 7
**T3**	ND	46 ± 9
**T4**	ND	0
**T25**	ND	30 ± 5
**T15**	ND	64 ± 8
**T26**	ND	64 ± 4
**T19**	ND	32 ± 8
**T22**	ND	0
**T28**	ND	64 ± 9
**T44**	ND	70 ± 2
**906**	ND	60 ± 8
**796**	36 ± 8	20 ± 1
**795**	ND	70 ± 7
**903**	ND	10 ± 1
**912**	ND	30 ± 8
**885**	100 ± 9	100 ± 9
**1253**	50 ± 8	ND
**Dm83**	69 ± 7	50 ± 8
**Ivermectin**	100	ND
**Amitraz**	100	100

ND: not determined.

**Table 5:** Toxicity and pharmacokinetic profile of compounds with the best acaricidal properties.

Chemical Collection Code	Mutagenicity by Ames Test	°LD_50_ (mg/kg)	Consensus Log Po/w	Solubility mg/mL	GI ** Absorption	•Log Kp cm/s	Metabolic Stability
**906**	Negative	5000	3.0	4 × 10^−2^	High	−6.3	Low
**796**	Negative *	>2000^+^	3.7	4 × 10^−2^	High	−5.3	Medium
**885**	Negative	2448	2.4	0.1	High	−5.7	Medium
**1253**	Positive	2015	1.4	0.2	High	−6.1	Low
**DM83**	Negative	1700	>5	<3 × 10^−4^	High	−4.9	Medium
**Amitraz**	Negative	400	4.8	1.6 × 10^−3^	High	−4.2	High

° Oral rat; • Skin permeation; * Ames test, experimental data; ^+^ in mice [26], ** gastrointestinal absorption.

## Data Availability

Not Applicable.

## Supplementary Materials

The following supporting information can be downloaded at: https://www.mdpi.com/article/10.3390/molecules27248863/s1; Table S1: Plant collection: general plant information, and extract production yields. Table S2: Data for the phenotypic screening. Table S3: Control percentage of the TIA in 8 ticks of the population of multiresistant field for compound 885 at 3 mM and Procedure for the synthesis of 2-methoxy-4-((E)-2-(5-((E)-ferrocenylvinyl)-1H-pyrazol-3- yl)vinyl)phenol (**Mar106**).
