# Supplementary material for: Phenotypic and Target-Directed Screening Yields New Acaricidal Alternatives for the Control of Ticks
Source: Molecules. 2022 Dec 13;27(24):8863. doi: 10.3390/molecules27248863 (PMC9781803; doi:10.3390/molecules27248863)
Supplement: Supplementary file 1 [file molecules-27-08863-s001.zip › molecules-1979398-Supplementary.pdf]

Table S1. Plant collection: general plant information, and extract production yields.

Common name	Scientific name	Location	Collection date	Part of the plant used	MeOH extract (g)	CH_2Cl_2 extract (g)	Initial sample (g)	Extraction Yield (%)
Bejuco de playa	*Ipomoea pes-caprae*	Tulum Beach (20°11'52.00"N; 87°26'12.38"O)	may-14	leaves and branches	20,2 (**T25**)*	2,4 (**T33**)	132	17
Nance	*Byrsonima crassifolia*	Ecological Park Chetumal (18°30'21.25"N; 88°19'12.79"O)	sep-13	tree bark	35,5 (**T27**)	12,6 (**T28**)	200	24
Uva de mar	*Coccoloba uvifera*	Chetumal bay (18°31'1.79"N; 88°16'14.46"O)	may-14	leaves	40,0 (**T24**)	4 (**T23**)	214	21
pucte, puk'te(maya).	*Terminalia catappa L.*	Chetumal city (18°31'0.55"N; 88°18'50.27"O)	may-14	leaves	27,9 (**T13**)	9 (**T6**)	250	15
				flowers	12,2 (**T34**)	nd(**T42**)	113	11
				fruit	27,9 (**T35**)	nd(**T41**)	362	8
Elemuy	*Malmea depressa*	Santa Rosa town (19°57'51.90"N; 88°16'17.00"O)	may-14	leaves and branches	25,6 (**T19**)	4	164	18
Elemuy	*Malmea depressa*	Santa Rosa town (19°57'51.90"N; 88°16'17.00"O)	may-14	root	9,5 (**T20**)	2,6 (**T21**)	187	6
Waxim	*Leucaena leucocephala*	Chetumal city (18°31'17.48"N; 88°18'47.94"O)	may-14	leaves and branches	26,8 (**T2**)	9 (**T8**)	273	13
Chaya	*Cnidoscolus chayamansa*	Chetumal city (18°31'0.55"N; 88°18'50.27"O)	may-14	leaves	16,7 (**T3**)	2 (**T4**)	100	19
Guarumbo	*Cecropia obstusifolia*	Chetumal city (18°31'26.60"N; 88°18'49.84"O)	may-14	leaves	16,7 (**T5**)	4,5 (**T22**)	150	14
sutup (maya).	*Helicteres baruensis Jacq.*	Tulum Beach (20°11'59.42"N; 87°26'53.68"O)	may-14	leaves and branches	10,6 (**T11**)	6 (**T14**)	113	15
habanero	*Capsicum chinense Jacq.*	F. Carrillo Puerto town (19°34'50.49"N; 88° 2'39.57"O)	may-14	fruit	11,3 (**T31**)	5 (**T38**)	55	21

* Nomenclature used for classifying the prepared extract.

Table S2. Data for the phenotypic screening.

Chemical collection code	Structure	% of growth inhibition
906	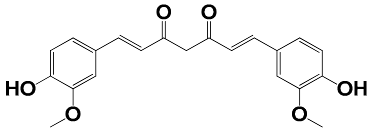	95
795	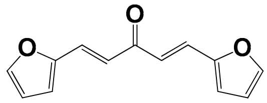	80
796	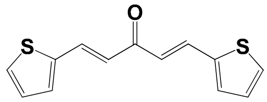	93
1019	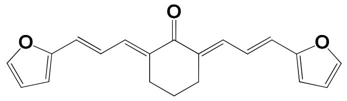	0
793	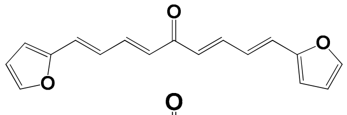	7
809	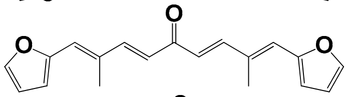	50
1223	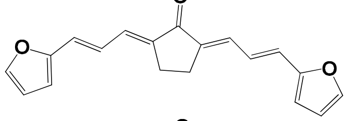	0
1018	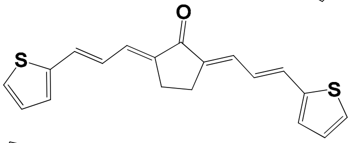	0
1140	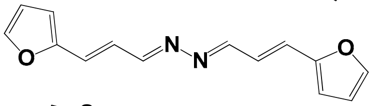	0
137	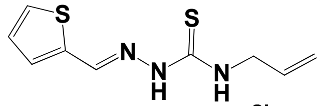	0
133	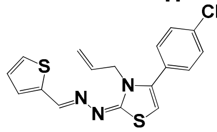	71
900	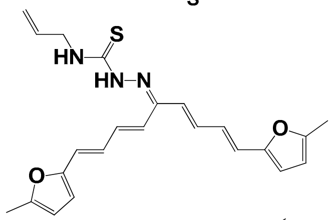	0
909	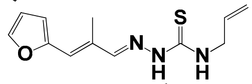	0
266	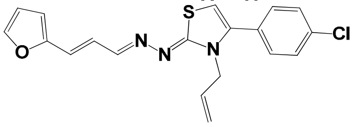	77

901	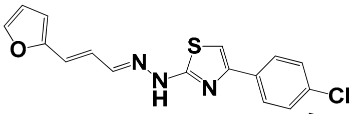	25
50	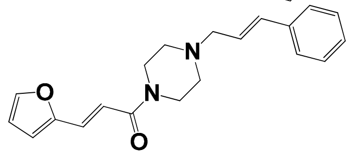	33
903	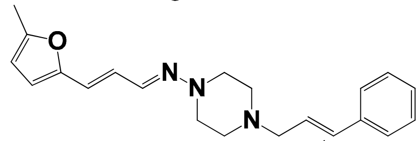	75
912	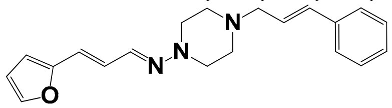	52
874	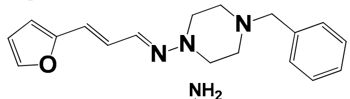	17
1105	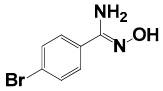	0
1125	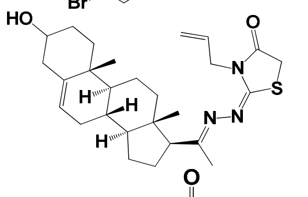	0
735	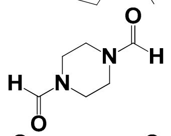	0
715	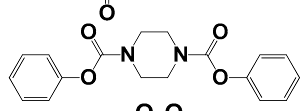	64
116	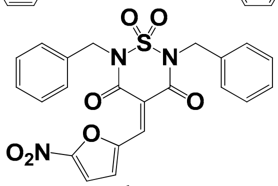	18
791	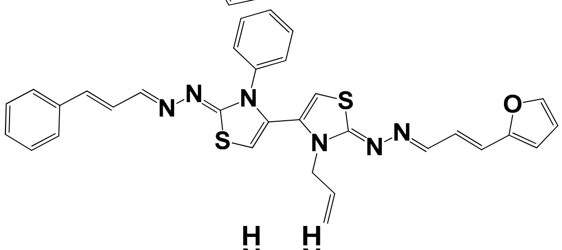	8
716	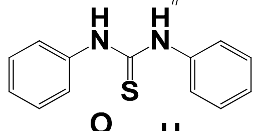	12
879	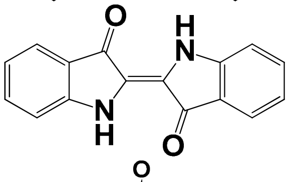	9
183	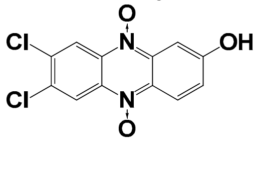	62

181	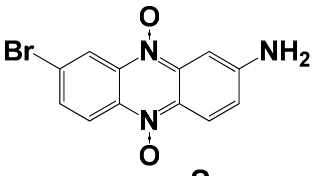	90
191	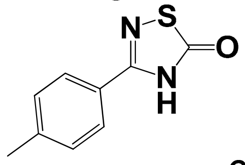	20
813	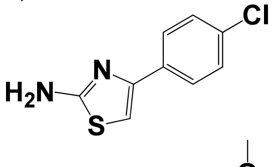	20
690	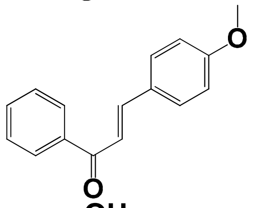	0
885	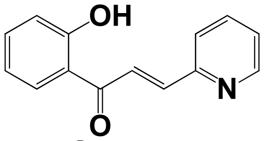	91
1253	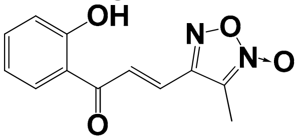	95

	Extract details	% of growth inhibition
1	*Croton spp.* (CH_2Cl_2)	19
2	*Leucaena leucocephala* (MeOH)	70
3	*Cnidoscolus chayamansa* (MeOH)	63
4	*Cnidoscolus chayamansa* (CH_2Cl_2)	100
5	*Cecropia obstusifolia* (MeOH)	0
6	*Terminalia catappa* (CH_2Cl_2)	2
7	*Pluchea spp.*	40

	(MeOH)	
8	*Leucaena leucocephala* (CH_2Cl_2)	72
9	*Byrsonima crassifolia* precipitated solid	26
10	*Ipomoea pes-caprae* precipitated solid	0
11	*Helicteres baruensis Jacg* (MeOH)	21
12	*Croton spp.* (MeOH)	2
13	*Terminalia catappa* (MeOH)	28
14	*Helicteres baruensis Jacg* (CH_2Cl_2)	0
15	*Ambrosia hispida* (MeOH)	93
16	*Amphipterygium adstringens* (MeOH)	0
17	*Byrsonima crassifolia* recrystallized solid	14
18	*Byrsonima crassifolia* second precipitated solid	12
19	*Malmea depressa* (MeOH)	63
20	*Malmea depressa* root (MeOH)	14
21	*Malmea depressa* root (CH_2Cl_2)	0
22	*Cecropia obstusifolia* (CH_2Cl_2)	100

23	*Coccoloba uvifera* (CH_2Cl_2)	0
24	*Coccoloba uvifera* (MeOH)	28
25	*Ipomoea pes-caprae* (MeOH)	70
26	*Ambrosia hispida* (CH_2Cl_2)	47
27	*Byrsonima crassifolia* (MeOH)	0
28	*Byrsonima crassifolia* (CH_2Cl_2)	49
29	*Pluchea spp.* (CH_2Cl_2)	9
30	*Aristolochia spp.* (MeOH)	14
31	*Capsicum chinese* (MeOH)	0
32	*Ruellia nudiflora (Engelm. & A. Gray) Urb.* (MeOH)	0
33	*Ipomoea pes-caprae* (CH_2Cl_2)	14
34	*Cassia fistula* flowers (MeOH)	0
35	*Cassia fistula* fruit (MeOH)	0
36	*Aristolochia spp.* (CH_2Cl_2)	0
37		0
38	*Capsicum chinese* (CH_2Cl_2)	0
39	*Amphipterygium adstringens* (CH_2Cl_2)	28
41	*Cassia fistula* fruit (CH_2Cl_2)	0

42	*Cassia fistula* flowers (CH_2Cl_2)	0
43	*Ruellia nudiflora* (CH_2Cl_2)	0
44	*Cassia fistula* leaves and branches (MeOH)	100

Table S3. Control percentage of the TIA in 8 ticks of the population of multiresistant field for compound 885 at 3 mM.

compound	% Control							
885	100	48	40	14	46	28	26	24

Procedure for the synthesis of 2-methoxy-4-((E)-2-(5-((E)-ferrocenylvinyl)-1H-pyrazol-3-yl)vinyl)phenol (Mar106).

A mixture of [(1E,6E)-7-(4-hydroxy-3-methoxyphenyl)-3,5-dioxo-1,6-heptadien-1-yl]ferrocene **Mar105** (150 mg, 0.34 mmol) and hydrazine hydrate (0.5 mL, 3.4 mmol) in glacial acetic acid (10 mL) was stirred at 70 °C for 3 h. The light red solution was then added to crushed ice (~100 mL). The resulting orange solid was filtered, washed with water (×3) and dried under vacuum and P_2O_5.

2-Methoxy-4-((E)-2-(5-((E)-ferrocenylvinyl)-1H-pyrazol-3-yl)vinyl)phenol (**Mar106**): Dark orange solid, Yield: 65 mg (52%); 1 H NMR (250 MHz, DMSO-d 6) δ: 3.83 (s, 3H, OCH 3), 4.15 (s, 5H, C 5 H 5), 4.32 (s, 2H, H-3 and H-4 of ferrocenyl), 4.54 (s, 2H, H-2 and H-5 of ferrocenyl), 6.59 (s, 1H, H-4 of pyrazole), 6.66-7.14 (m, 7H, vinylic and aromatic protons), 9.16 (s, 1H, NH), 12.76 (OH).; 13 C NMR (62.5 MHz, DMSO-d 6) δ: 55.6, 66.7, 67.7, 68.3, 69.0, 79.2, 99.0, 109.4, 109.5, 110.7, 115.6, 118.4, 120.0, 128.4, 131.3, 146.4, 146.9, 147.7, 147.9, 152.8.
